# The Development of *Macrophomina phaseolina* (Fungus) Resistant and Glufosinate (Herbicide) Tolerant Transgenic Jute

**DOI:** 10.3389/fpls.2018.00920

**Published:** 2018-07-10

**Authors:** Shuvobrata Majumder, Karabi Datta, Chirabrata Sarkar, Subhas C. Saha, Swapan K. Datta

**Affiliations:** ^1^Laboratory of Translational Research on Transgenic Crops, Department of Botany, University of Calcutta, Kolkata, India; ^2^Quality Assurance Section, ICAR-National Institute of Research on Jute and Allied Fibre Technology, Kolkata, India

**Keywords:** FRHT jute, fungus resistant, herbicide tolerant, *Macrophomina phaseolina*, stem rot, glufosinate ammonium, bast fiber, transgenic jute

## Abstract

The worldwide demand for natural bast fibers is met aptly by the long, golden and silky fibers of jute. This highest bast fiber producing crop is of great applicability and is extensively used in paper and textile industry. *Macrophomina phaseolina* (Tassi) Goid is a severely devastating necrotrophic fungal pathogen causing stem rot, root rot, and charcoal rot diseases in both the cultivated species of jute – *Corchorus capsularis* and *Corchorus olitorius*. Another major problem faced in jute cultivation is profuse weed infestation in the fields. Huge losses in quality fiber production is caused by this pathogenic fungi and cultivation cost increases as well due to weed management expenditure during cropping season. To solve these long persisting jute cultivation challenges, the chitinase (*chi11*) gene (to provide fungus resistance) and the *bar* gene (to provide herbicide tolerance) have been incorporated in *C. capsularis* JRC-321 via *Agrobacterium* transformation and analyzed up to T_2_ generation. Stable integration and expression of these two genes in the jute genome was confirmed upon extensive analyses. Transgenic plants showed higher chitinase expression and chitin degrading activity than non-transgenic control plants. Antifungal activity significantly increased in transgenic plants as confirmed by detached leaf and whole plant *M. phaseolina* bioassay. Herbicide tolerance was analyzed by growing transgenic plants in 10 mg/l glufosinate ammonium containing media and by spraying 0.25% (v/v) glufosinate herbicide Basta^®^ on them. Assessment of residual phytotoxicity effects of Basta^®^ on soil confirmed no negative impact on growth of indicator plants corn and cucumber. Transgenic jute plants were at par with non-transgenic (control) jute plants in all phenotypic aspects. Non-transgenic (control) jute plants suffered significant losses in fiber yield and quality due to *M. phaseolina* infection whereas the transgenic lines maintained the quality of fiber even after the infection.

## Introduction

Jute (*Corchorus* sp.) plants provide long, golden and natural bast fibers that are applied for various industrial and domestic uses. The lignocellulosic fibers of jute are the second most economically important fibers after cotton. Out of more than 170 species of jute, only *C. capsularis* and *C. olitorius* are cultivated abundantly in South Asian countries. About 99% of the total global jute production was from the developing countries of Asia, where India contributed 56.85%, Bangladesh 40.67%, China 1.03%, Uzbekistan 0.61%, and Nepal 0.45% (FAO^1^). Jute farmers face a variety of difficulties during cultivation, two of them being plant diseases and unwanted weeds or herbs in the field. Diseases like seedling blight, leaf blight, leaf mosaic, charcoal rot, stem rot, root rot, and anthracnose almost completely damage crops. *Macrophomina phaseolina* (Tassi) Goid, a necrotrophic fungal pathogen, causes the stem rot, root rot and charcoal rot diseases of jute in both the cultivated varieties (Meena et al., 2015). *M. phaseolina* infects more than 500 different plant species of more than 100 plant families including major food crops (maize and sorghum), pulse crops (green gram, mung bean, groundnut, and sesame), oil crops (sunflower, soybean, and castor) vegetable crops (tomato, potato, onion, and garlic) and fiber crop (cotton) (Wyllie, 1988; Das et al., 2008). It remains infectious for more than 4 years in soil and crop residue as sclerotia (Islam et al., 2012). Under suitable conditions, hyphae germinate from the sclerotia and infect the crop plant by penetrating the cell wall. Losses in jute yield, caused by *M. phaseolina*, were recorded to be 35–40% in India and up to 30% in Bangladesh (Roy et al., 2008; Islam et al., 2012). Till date, no cultivable jute variety, that has complete resistance against *M. phaseolina*, has been reported by conventional breeding approaches.

The hot (20–40°C) and humid (70–80%) climate coupled with intermittent rainfall (50–80 mm), that favor jute cultivation, also encourage profuse weed growth in the field, resulting in severe crop-weed competition for soil nutrition and ultimately causing up to 70% fiber yield loss (Kumar et al., 2013). Losses could be more if sufficient care is not taken in the first 15 to 45 days after sowing (DAS), which is the “critical period” for weed control for jute cultivation (Kumar et al., 2013). Losses continue when investment in weed management increase the total jute cultivation cost by more than 35% in India and 30–40% in Bangladesh (Kumar et al., 2013; Islam, 2014). Presence of about 129 species of weeds were reported in jute fields in Asia (Islam, 2014).

Chemical control of weed management, by applying herbicides, is a popular practice along with other methods as listed by Kumar et al. (2013) and Islam (2014) on jute cultivation. Based on “time of application” three categories of herbicides are used in jute fields: pre-planting, pre-emergence and post-emergence herbicide. Pre-planting herbicides – uchloralin, trifluralin, and EPTC (thiocarbamate) are applied from 7 days before sowing to 3 days after sowing of jute, for management of annual grass and broadleaved weeds. Pre-emergence herbicides are applied immediately or 1–2 DAS of jute and before the emergence of weed and jute plantlets. Some examples are pendimethalin, *s*-metolachlor, butachlor, trifluralin, and pretilachlor. Post-emergence herbicides are applied 15–30 DAS and after the emergence of jute plantlets and weeds. Some examples are cyhalofop, fenoxaprop ethyl, and quizalofop ethyl. Based on “selectivity,” two categories of herbicides – selective and non-selective, are used in jute fields. Selective herbicides are also called narrow spectrum herbicides that kill a particular group of plants. In jute fields 2,4-D selectively kill broadleaf weed plants from a mixed population of jute plants and weeds. Alternatively, non-selective herbicides or broad spectrum herbicides kill indiscriminately, both weeds and crops alike. Some examples are paraquat, glyphosate (Roundup^®^) and glufosinate (Basta^®^). Weed management by chemical herbicides is more cost-effective than alternative weed control methods that includes hand weeding, sawdust mulches and sowing cover crops in fields (George and Brennan, 2002; Ghorai, 2008). Although broad spectrum herbicides are preferred over selective ones, it could induced “crop injury” during field application. Therefore introducing herbicide tolerance (HT) in such crop plants is highly desirable when weed infestation is a major challenge.

In this study, fungus resistant (FR) transgenic jute plants were developed by introducing the *rice chitinase* (*chi11*) gene in the jute (*C. capsularis*, variety JRC-321) genome. The *rice chitinase* (*chi11*) is the most studied chitinase gene of plant origin, that breaks fungal chitin by hydrolysis of the β-(1, 4) linkages of chitin found in fungal cell wall (Cletus et al., 2013). Since about 60% of fungal cell wall consists of chitin, chitinase cause major damage to fungus (Bartnicki-Garcia, 1968). Constitutive overexpression of the rice chitinase in transgenic plants such as in rice (Datta et al., 2000, 2001; Karmakar et al., 2017), wheat (Chen et al., 1998), cucumber (Kishimoto et al., 2002), strawberry (Asao et al., 1997), rose (Marchant et al., 1998), and grapevine (Yamamoto et al., 2000) enhances resistance to different fungal pathogens other than *M. phaseolina*. Work on *M. phaseolina* resistant transgenic plant development is scarce, only transgenic potato with Thaumatin-like proteins (TLPs) of *Camellia sinensis* (*CsTLP*) by Acharya et al. (2013) and transgenic tobacco with Pathogenesis-related (PR) gene from *Jatropha curcas* (*JcPR10a*) by Agarwal et al. (2016) have been reported. Sharma et al. (2014) found that upon infection by *M. phaseolina*, in a resistant variety of sorghum (PJ-1430), the expression of sorghum chitinase was induced within 0 and 24 h and in case of susceptible variety (SU-1080) induction was in 48 h. The expression levels of chitinase in resistant cultivar were significantly higher and faster than the susceptible sorghum variety. This resistance to *M. phaseolina* by chitinase fueled the concept of FR transgenic jute plants.

An assortment of genes are being used for HT crop development and approved worldwide for commercial use, some examples are – *epsps* against glyphosate, *bar* and *pat* against glufosinate, *aad-1* and *aad-12* against 2,4-D, *hppd* against isoxaflutole, *bxn* against oxynil and *als* against sulfonylurea herbicide^2^. In this study the *bar* (*b*ial*a*phos-*r*esistance) gene, cloned from *Streptomyces hygroscopicus*, was used for HT jute development. The *bar* gene, encoding phosphinothricin acetyltransferase (PAT), provides resistance to glufosinate ammonium or phosphinothricin (PPT) or glufosinate (Datta et al., 1992). PPT is the principle ingredient of commercially available herbicides like Basta^®^ (13.50–18.02%), Buster^®^ (20.00%), Finale^®^ (11.33%), and Liberty^®^ (10–24.50%).

In this investigation, our objectives were (i) *Agrobacterium* mediated shoot tip transformation of *chi11* and *bar* genes into the jute genome; (ii) analysis of fungus resistance quality of transgenic jute plants against *M. phaseolina* and assessment of fiber quality under conditions of fungal infection; (iii) analysis of herbicide tolerance ability of transgenic jute plants against a glufosinate herbicide.

## Materials and Methods

### Construction of Plant Transformation Vector

**FIGURE 1:**
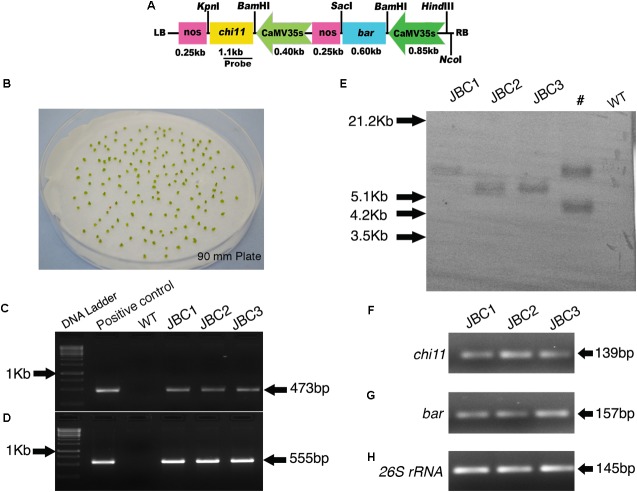
Generation of transgenic plants, screening and integration of transgenes in T_0_ plants. **(A)** Schematic map of *pCAMBIA1301-bar-chi11* transformation vector. **(B)** Shoot tips after 3 days of co-cultivation with *A. tumefaciens* (LBA4404). **(C)** PCR analysis of T_0_ transformants and positive control showing partial amplification of 473 bp *chi11* gene and **(D)** 555 bp *bar* gene. No amplification in control (WT) plants. **(E)** Southern blot analysis of genomic DNA of T_0_ plants digested with *Nco*I and probed with DIG labeled 473 bp of *chi11*. #Not applicable for investigation. Semi quantitative RT-PCR gel picture of 3 T_0_ transgenic plants. **(F)** for *chi11* gene **(G)** for *bar* gene and **(H)** for jute internal control *26S rRNA* gene.

To construct the *pCAMBIA1301-bar-chi11* gene cassette, the rice *chitinase* gene *chi11* (1.1 kb) was cloned from the vector *pGL2* (Lin et al., 1995) and the *bar* gene (0.55 kb) was cloned from the *pGPTV-bar/Fer* vector (Vasconcelos et al., 2003). The binary vector *pCAMBIA1301-bar-chi11* was prepared using the cloning vectors pUC19 and pTZ57R/T. The *pUC19-CaMV35S-chi11-nos* gene cassette was developed by cloning the rice *chitinase* gene *chi11* (1.1 kb) under a 0.40 kb *CaMV35S* promoter and a 0.25 kb nopaline synthase (*nos*) terminator in a pUC19 cloning vector. The *bar* gene (0.55 kb) was cloned in pTZ57R/T vector using INSTACLONE PCR CLONING KIT^®^ (Thermo Scientific, Waltham, MA, United States) and *bar* gene specific primers (**Table 1**). The presence of *bar* gene was confirmed by DNA sequencing of pTZ57R/T-*bar* vector followed by sequence alignment with the help of BLAST^TM^ program^3^. Then the *bar* gene was removed by *Bam*HI-*Sac*I restriction enzymes (NEB, Ipswich, MA, United States) from the pTZ57R/T-*bar* vector and cloned under 0.85 kb *CaMV35S* promoter and 0.25 kb *nos* terminator to develop *pUC19-CaMV35S-bar-nos* gene cassette. Plant transformation binary vector pCAMBIA1301 (Accession No. AF234297) was modified by removing its *β-glucuronidase* (*gus*) reporter gene after digestion with *Bst*EII–*Bgl*II restriction enzyme and its *hygromycin phosphotransferase* (*hpt*) marker gene was removed after *Xho*I (NEB) digestion. The *CaMV35S-bar-nos* gene cassette (1.7 kb) was removed from *pCU19-CaMV35S-bar-nos* cloning vector and inserted into modified pCAMBIA1301 in *Hind*III-*Eco*RI site to develop *pCAMBIA1301-CaMV35S-bar-nos* vector initially. Then the *CaMV35S-chi11-nos* gene cassette (1.75 kb) was removed from *pUC19- CaMV35S-chi11-nos* cloning vector by *Hind*III-*Eco*RI followed by ligation of *CAMBIA1301-CaMV35S-bar-nos* vector to develop the *pCAMBIA1301-CaMV35S-bar-nos-CaMV35S-chi11-nos* transformation vector. This vector construct has been abbreviated as *pCAMBIA1301-bar-chi11* (**Figure 1A**). Orientation of genes in *pCAMBIA1301-bar-chi11* vector was confirmed by nucleotide sequencing and transformed into *Agrobacterium* strain LBA4404.

**Table 1:** List of primers used in this investigation (Designed by PrimerQuest^®^ software and synthesized through Integrated DNA Technologies, United States).

Gene (accession number)	Primer name	Primer sequence (5^′^ to 3^′^)	Amplicon size and anneling temperature	Use of PCR amplified product
Rice *chitinase* (X54367)	CHI-473-F	TCGCCTCCATCATATCGCCCTC	473 bp and 53.0°C	PCR-screening, Probe preparation in Southern
	CHI-473-R	CGTCATCCAGAACCAGAACGCC		hybridization
	RT-CHI-F	GTTCTGGTTCTGGATGACGC	139 bp and 59.0°C	RT-PCR, qRT-PCR
	RT-CHI-R	GCCGTTGATGATGTTGGTGA		
*bar* (JQ293091)	BAR-555-F	CGCCGATGGTTTCTACAAAGA	555 bp and 60.5°C	PCR-screening, Vector construction
	BAR-555-R	TCAATGACCGCTGTTATGCG		
	RT-BAR-F	CTACACCCACCTGCTGAAG	157 bp and 59.0°C	RT-PCR, qRT-PCR
	RT-BAR-R	CCAGTTCCCGTGCTTGAAG		
Jute *26S rRNA* (JK743816)	RT-26S-F	GTTCCACACGAGATTTCTGTTC	145 bp and 59.0°C	RT-PCR, qRT-PCR (as internal control)
	RT-26S-R	TTTTAGACCCAAGACCGGC		

### Preparation of Plant Material and Explant

Central Research Institute for Jute and Allied Fibres (CRIJAF), Indian Council of Agricultural Research, Kolkata, India provided the seeds of *C. capsularis* (variety JRC-321). Surface sterilization of jute seeds was done by immersing in 70% ethanol for 5 min, followed by shaking in a mixture of sodium hypochlorite and Tween 20^®^ for 20 min and then rinsing 4–5 times in sterile water. A “germination” media [constituted by MS salts and vitamins (HiMedia, Laboratories, Pvt. Ltd., Mumbai, India) + 1.5% sucrose + 0.8% agar at pH 5.8] was used to grow the seeds in culture room (28°C, 16 h light and 8 h dark photoperiod). Seedlings of 8–12 day-old were dissected to isolate shoot tips for transformation in sterile condition.

### Transformation and Regeneration of Jute Plants

*Agrobacterium* mediated shoot tip transformation and *in vitro* regeneration of explants were done as described by Majumder et al. (2018). Isolated shoot tips were immersed in the *Agrobacterium* “infiltration/co-cultivation” media [MS salts and vitamins + 0.1% myo-inositol + 2% sucrose + 0.5 mg/l N6-benzylaminopurine (BAP) + 1 mg/l indole-3-acetic acid (IAA) + 0.2 mg/l gibberellic acid (GA_3_) + 20 mg/l acetosyringone at pH 5.6] for 1 h in dark. Suspension media contained about 1.380 × 10^8^ cells/ml (OD 0.3 at 600 nm) *Agrobacterium* (LBA4404) cells harboring the *pCAMBIA1301-bar-chi11* construct. Incubated shoot tips were vacuum infiltrated under a pressure of 600 mm Hg for 10 min followed by soaking and placing on Whatman No. 1 filter paper with their apices upward. Finally “co-cultivation” media was added, sealed and kept at 28°C in dark for 72 h. The co-cultivated shoot tips were washed 3–4 times in “Agrobacterium-washing” media (MS salts and vitamins + 1.5% Sucrose + 500 mg/l Timentin at pH 5.6) (**Figure 1B**). Shoot tips were transferred to “shoot tips elongation/selection” media (MS salts and vitamins + 0.1% myo-inositol + 3% sucrose + 1 mg/l BAP + 0.5 mg/l IAA + PPT + 0.8% agar at pH 5.8). Transformed shoot tips were selected by three consecutive selections at fortnightly intervals on 1.0, 2.5, and 4.0 mg/l PPT (PESTANAL^®^, Sigma-Aldrich, St. Louis, MO, United States) supplemented media. Selected shoot-tips were transferred to “rooting” media [half strength MS salts and vitamins + 0.05% myo-inositol + 1.5% sucrose + 0.3 mg/l indole-3-butyric acid (IBA) + 0.6% agar at pH 5.8] without any selection pressure for rooting. Finally, regenerated plants were transferred to soil for establishment in the greenhouse.

### Chlorophenol Red (CPR) Assay for Qualitative Detection of *bar* Gene

Assay was performed according to Wright et al. (1996) with some modifications. Leaves were weighed (25 mg), surface sterilized and submerged in 5 ml CPR assay media containing 10 mg/l PPT in 35 mm petri plates. The petri plates were then sealed with PARAFILM^®^ and kept in culture room conditions for 48 h. The test results were derived from leaf samples that changed color to either yellow/orange (positive) or red/purple (negative). The pH of the media was measured. All progeny plants of the three transgenic lines were screened by CPR assay with three technical replcations.

### Genomic DNA Extraction and PCR Screening

Genomic DNA was isolated from 100 mg young opened leaves using the NUCLEOSPIN PLANT II^®^ KIT (Macherey-Nagel GmbH & Co. KG, Neumann Neander Straße, Germany). The T_0_ plants and the progeny plants of subsequent generations (T_1_ and T_2_) were screened for *chi11* and *bar* genes using specific primers in PCR with 100 ng genomic DNA (**Table 1**). The amplified products were subjected to electrophoresis in 1.5% (w/v) agarose gel.

### Southern Hybridization Analysis

Southern hybridization was carried out according to Sambrook and Russell (2001). After digestion of 15 μg genomic DNA with *Nco*I (NEB), it was separated in 1% agarose gel and transferred to a positively charged nylon membrane. As per manufacturer’s instruction (Roche, Basel, Switzerland), a probe (473 bp of *chi11* PCR product) was labeled with digoxigenin (DIG)-dUTP and detected by DIG DNA labeling and detection kit.

### RT-PCR and qRT-PCR Analysis of Transgenic Plants

Total RNA was isolated from transgenic and WT control jute plants by using NUCLEOSPIN RNA PLANT^®^ isolation kit (Macherey-Nagel). The cDNA synthesis was accomplished using 1 μg of total RNA in iSCRIPT RT SUPERMIX^®^ cDNA synthesis kit (Bio-Rad, Hercules, CA, United States) following manufacturer’s instructions. The qRT-PCR was performed in CFX 96 Real time system^®^ (Bio-Rad), in triplicates. Jute *26S ribosomal RNA* (*26S rRNA*) gene was used as internal control to normalize all data (Majumder et al., 2018). The qRT-PCR reaction mixture was constituted with specific primers (**Table 1**), MAXIMA SYBR GREEN^®^ (Thermo Scientific) and cDNA. Quantitative variation among different samples was determined using the 2^-ΔΔCT^ method. All the data were analyzed using CFX MANAGER^®^ software (Bio-Rad). The cDNA was also used in semi-quantitative RT-PCR for partial amplification of *chi11* and *bar* genes. The RT-PCR and qRT-PCR analyses were performed with 10 biological replicates from each T_1_ transgenic line (JBC1, JBC2, and JBC3) with three technical replications for each plant.

### Gel Diffusion Assay for Visualization of *chi11* Activity

The gel diffusion assay was performed according to Zou et al. (2002) with modifications. Gel plate was made by solidifying gel consisting of 1.5% agarose with 0.4% colloidal chitin at pH 5.0. Upon solidification a 5 mm diameter cork borer was used to make wells in the gel. Equal amounts (150 μg) of extracted crude protein was pipetted into each well. The loaded sample plate was sealed and incubated at 37°C for 24 h. After incubation, plate was immersed in staining solution containing 0.1% calcofluor white (Fluka-Sigma, St. Louis, MO, United States). Lytic zones in the gel plate were visualized by UV transillumination and photographed under a Gel Documentation System with Image Lab^®^ software (Bio-Rad). Area of dark ring zone was measured by placing each on graph paper. Data were recorded from three independent experiments for each sample. A total of 10 biological replicates were analyzed from each transgenic line.

### In-Solution Activity Assay of Chitinase

Chitinase activity assay was performed as described by Karmakar et al. (2015). Total protein was isolated from 50 to 60 DAS transgenic and WT leaves (100 mg) using 0.1 M sodium phosphate buffer (pH 5.0). Equal amounts (100 μg) of total protein were incubated with colloidal chitin at 50°C for 1 h. The resulting product *N*-acetyl glucosamine residue was spectrophotometrically measured at 530 nm using dinitrosalicylic acid (DNSA) method. Experiment was performed with 10 biological replicates from each transgenic line and three technical replications for each plant.

### Qualitative ELISA for PAT Enzyme

A qualitative sandwich enzyme-linked immunosorbent assay (ELISA) was performed using QualiPlate^TM^ (EnviroLogix, Portland, ME, United States) to confirm the presence of PAT/*bar* in crude protein extracted from young-opened leaves (50 mg) of 50–60 DAS transgenic plants. Experiment was performed with 20 biological replicates from transgenic line (JBC1, JBC2, and JBC3) with two technical replications for each sample (leaves) according to manufacturer instructions. Absorbance was measured at 450 nm in a microplate reader (Bio-Rad).

### Ammonia Assimilation (AA) Assay for Qualitative Estimation of *bar* Gene Expression

**FIGURE 2:**
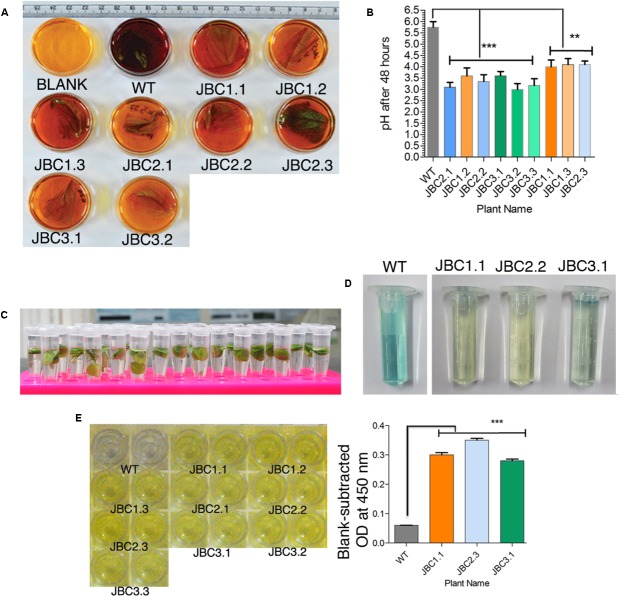
Qualitative analysis specific to *bar* gene and its functionality. **(A)** Screening of transgenics by CPR assay. After incubation, media (with 10 mg/l PPT) color changed to yellow-orange for transgenic leaf (with *bar* gene) and red-purple for *bar* negative WT plant leaf. Here the transgenic plants JBC1.1, JBC1.2, and JBC1.3 represent the JBC1 line, JBC2.1, JBC2.2, and JBC2.3 represent the JBC2 line, and JBC3.1 and JBC3.2 represent the JBC3 transgenic line. **(B)** The *bar* positive leaf containing media showing pH range of 3.20–4.20 with significant difference (^∗∗^*P* = < 0.01, ^∗∗∗^*P* = < 0.001) as compared to WT by one-way ANOVA at *P* < 0.05 and Tukey’s multiple comparisons tests using Graphpad Prism 6 software. **(C)** In ammonia assimilation assay leaf disks (5 mm) incubated in 10 mg/l PPT containing incubation media for 8 h in light. **(D)** Change in color of the solution judged as either positive (pale blue to yellow color) or negative (dark blue color) plants. Here, JBC1.1 transgenic plant represent the JBC1 line, JBC2.2 represent the JBC2 line and JBC3.1 represent the JBC3 transgenic line. **(E)** Yellow color developed in positive wells of QualiPlate ELISA plate. OD measured at 450 nm and significant difference (^∗∗∗^*P* = < 0.001) found between transgenic and WT samples by Tukey’s multiple comparison tests. OD ≥ 0.2 is considered as PAT/*bar* positive. Here, specific individual plants are the representatives of the three transgenic lines JBC1, JBC2, and JBC3.

Assay was performed according to Deblock et al. (1995) with some modifications. Five leaf disks (5 mm each) were isolated using cork borer and incubated in 1 ml incubation media (50 mM potassium phosphate buffer pH 5.8, 2% sucrose, 0.1 mg/1 2,4-D, 0.1% Tween 20, and 10 mg/l PPT) for 8 h under light (**Figure 2C**). Finally the solution was judged qualitatively as either *bar* positive or negative by visual observation of developed color. Experiment was performed with 10 biological replicates from each of the three transgenic lines (JBC1, JBC2, and JBC3) with three technical replicates for each sample (leaf).

### Anti-fungal Bioassay of Detached Leaf by *M. phaseolina*

The bioassay of detached leaves, from transgenic and control jute plants, was performed with the help of mycelia agar disks. Mycelia disks were cut out from the peripheral region of petri plate of 5-day-old *M. phaseolina* culture (**Figure 7A**), grown on potato dextrose agar media (HiMedia, Laboratories, Pvt. Ltd., India). In a 140 mm petri plate, three leaves from each plant were placed on 2% agar after surface sterilization. A mycelial agar disk, of 5 mm diameter, was placed on each leaf (**Figure 7A**). The lid was sealed with Parafilm^®^ and kept at 28°C for 72 h in dark. Area of necrosis (dark brown-black color zone) was marked and measured by using ‘Analysis’ measurement tool in Photoshop^®^ CS3 software (Adobe, United States). The necrosis area of WT plant was assigned 100% and the level of disease resistance percentage was calculated as the ratio of necrosis in the independent transgenic lines relative to WT (according Chen et al., 2014). Three independent experiments were done for each plant.

### Whole Plant Antifungal *M. phaseolina* Bioassay

Plants of about 80 to 90 DAS and of similar heights were selected for whole plant fungus bioassay. A 5 mm diameter *M. phaseolina* mycelial agar disk was placed on the pricked stem region and covered with moist sterile cotton (**Figure 8B**). The cotton covering was removed after 48 h from all such plants. Bioassay was carried out for 20 days. Lesion lengths of infected stems were measured and mean lesion length for individual plant was calculated.

### Analysis of Transgene Segregation by Growing T_2_ Transgenic Seeds in 10 mg/l PPT Containing MS Media

Transgenic seeds of advanced progeny (T_2_ generation) were surface sterilized and incubated at 37°C and kept overnight in dark conditions on moist filter paper to initiate imbibition. Two hundred imbibed seeds (with emerged radical) were distributed evenly in 78 mm round Phyta jars (HiMedia, Laboratories, Pvt. Ltd., Mumbai, India) containing 10 mg/l PPT supplemented MS media. After 10 days the number of PPT resistant (healthy plants only) and PPT sensitive seedlings were recorded. The ratio of PPT resistant to PPT sensitive was compared with Mendelian segregation 3:1 ratio by Chi square test.

### Whole Plant Bioassay by Glufosinate Herbicide Basta^®^

This herbicide tolerance bioassay was carried out in two-ways – planting transgenic and WT plants in ‘different pots’ and in ‘same pot.’ In the ‘different pots’ analysis, five randomly selected T_1_ progeny seedlings, from each independent transgenic line, were grown in individual pots (254 mm diameter). At 30 DAS, transgenic and WT plants were bioassayed by spraying 0.25% (v/v) (0.84 mg/l of PPT) Basta^®^ (Bayer Cropscience, Ltd., Mumbai, India). Bioassay was carried out for 10 days in greenhouse condition. The ‘same pot’ assay was performed in a large pot (410 mm diameter) where one half was used for transgenic (progenies of any particular line) and in other half WT seeds were sown, keeping a separable gap in between. Bioassay was carried out on 50–60 mm seedlings by spraying 0.25% (v/v) Basta^®^. After 10 days the number of surviving plants were noted for each transgenic line.

### Assessment of Residual Phytotoxicity Effects of Basta^®^ on Soil

The potted (254 mm diameter) soil was treated by spraying 0.25, 0.50, and 1.0% (v/v) Basta^®^ herbicide. The phytotoxicity of soil was checked by the help of indicator plants- cucumber and corn. After 6 days of Basta^®^ spray, 10 seeds each of cucumber and corn were planted in separate designated pots. In a duplicate setup, equal number of seeds were sown in non-treated pots (where only water was sprayed) for experimental control. Seed germination (seedling count) was recorded after a week and plantlets were observed for up to 2 weeks after sowing for any noticeable phenotypic changes in order to determine residual effect of Basta^®^ in the soil. Measurements were taken for plant height, from soil surface to tip of the youngest leaf for corn plants and to the point of growth for cucumber plants. Experiment was done in replicates of three.

### Assessment of Morphology and Phenotype

Randomly selected, 10 greenhouse grown transgenic progeny plants of T_2_ generation (i.e., 10 biological replicates for the experiment) from each line (i.e., JBC1, JBC2, and JBC3) and wild type (WT) jute plants of age 110–120 DAS were compared by measuring for full plant height, stem length, and basal stem diameter.

### Assessment of Fiber Retting and Quality of Jute Fiber

Fungus bioassayed T_1_ transgenic progeny plants, at 120–130 DAS, were biologically retted at 28–34°C. Fibers were extracted, sun dried and recorded for length (m), fiber strength (g/tex), and fiber fineness (tex). Transgenic progeny plants, from the three transgenic lines (JBC1, JBC2, and JBC3), were categorized on the basis of their lesion length after whole plant fungus bioassay as – very high tolerance (VHT), high tolerance (HiT), moderate tolerance (MT), and low tolerance (LT). Fiber quality was analyzed from plants from each category.

### Statistical Analyses

The Graphpad Prism 6 software^4^ was used to perform all statistical analyses. The experimental data are presented as mean ± standard error (SE) for the three or more replicates. ANOVA was used to compared the means and differences between group means keeping statistical significance (*P* < 0.05) and Tukey’s multiple comparisons under consideration. Chi-square test for goodness-of-fit was done by using Graphpad Prism online tool^5^.

## Results

### Generation of Transgenic Plants, Screening, and Integration of Transgenes

Shoot tips were co-cultivated with *Agrobacterium* cells that harbored *chi11* and *bar* genes. The *chi11* and *bar* genes contained transformation vector *pCAMBIA1301-bar-chi11* (**Figures 1A,B**). A total of 10 T_0_ transgenic plant lines were developed with 3% transformation efficiency and these independent transgenic lines were named as JBC (**j**ute with ***b**ar* and ***c**hi11*) 1, 2, and 3, respectively (Supplementary Table 1). Shoot tips were grown in PPT supplemented MS selection media in gradually increasing concentration of 1 to 4 mg/l PPT (w/v) (Murashige and Skoog, 1962). Putative transgenic plants were grown in greenhouse. T_0_ putative transgenic plants were screened based on PCR for the presence of *bar* and *chi11* genes. After PCR, specific amplifications of 473 bp for *chi11* (**Figure 1C**) and 555 bp for *bar* genes (**Figure 1D**) were visualized in agarose gel. No such amplifications were observed for non-transgenic control (WT) jute plants. Southern blot analysis of PCR positive T_0_ plants (JBC1, JBC2, and JBC3) confirmed the single locus integration of *pCAM-bar-chi11* vector (**Figure 1E**) as reported here. Southern blot and PCR analysis confirmed the successful integration of the T-DNA of *pCAMBIA1301-bar-chi11* transformation vector in *C. capsularis* (variety JRC-321). The *chi11* and *bar* gene were present in the same T-DNA. Therefore integration of *chi11*, confirmed by southern analysis, also established the integration of *bar* in transgenic jute lines. No transgene integration was found in non-transgenic WT control plants. Integration of *pCAM-bar-chi11* vector was also confirmed in reverse transcriptase PCR (RT-PCR) by amplifying *chi11* and *bar* genes from the cDNA of T_0_ transgenic plants (**Figures 1F–H**). RT-PCR analysis confirmed the production of mRNA transcripts by *chi11* and *bar* genes in transgenic plants.

### Qualitative Analysis of *bar* Gene

Presence of *bar* gene and its functionality were tested and confirmed in T_0_ and subsequent T_1_ and T_2_ transgenic generations. In CPR assay, transgenic plants were subjected to confirmation test for the presence of *bar* gene by the pH indicative property of chlorophenol red (**Figure 2A**). After 48 h incubation period, color of the 10 mg/l PPT containing liquid MS media changed from yellow to dark reddish-purple with pH > 4.25 for the negative (without *bar*) plants. The color of media for positive (*bar* containing) plants were much lighter to unchanged with the mean pH range 3.20–4.20 (**Figure 2B**). Significant difference of mean (at *P* < 0.05) was found in the pH levels of *bar* positive, negative (segregated progeny) and non-transgenic WT control plants after one-way ANOVA analysis of the pH data. The significant difference (*P* ≤ 0.01) in pH values of WT and transgenic plants was found in Tukey’s multiple comparison test (**Figure 2B**).

In AA assay the diffused ammonia, in 10 mg/l PPT containing incubation media, was chemically detected along with color development (**Figure 2C**). Yellow (JBC1.1 and JBC2.2) to pale blue (JBC3.1 and JBC2.2) color was observed for positive plants with the *bar* gene and dark blue color was observed for WT control and negative plants (**Figure 2D**).

In PAT/*bar* ELISA analysis, blank-subtracted OD at 450 nm for all transgenic plant samples were in the range 0.24 to 0.35 (**Figure 2E**) and WT showed OD < 0.06. This difference in OD values was found to be statistically significant (*P* < 0.001) in one-way ANOVA analysis and Tukey’s multiple comparison test (at *P* < 0.05). According to manufacturer guidelines for this ELISA kit, OD at 450 nm ≥ 0.2 is considered to be a PAT/*bar* positive.

**FIGURE 3:**
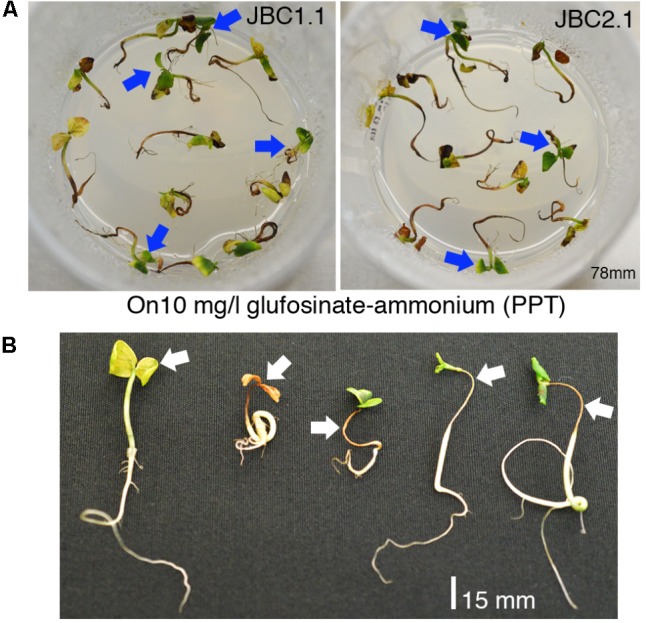
Selection of T_1_ seedlings in 10 mg/l glufosinate ammonium. **(A)** Positive seedlings successfully grown in high dose of PPT (Blue arrows). **(B)** The negative plants (White arrows), showing browning of stem and root, rapid degradation of cotyledon chlorophyll, no lateral root formation, and necrosis.

The presence and inheritance pattern of *bar* gene, in T_2_ transgenic progenies, were checked from the surviving percentage of plants in PPT (10 mg/l) supplemented MS media. Distinct phenotypic differences were observed between the transgenic progeny plants 5–6 days after germination (**Figure 3A**). The *bar* negative (PPT sensitive) plants showed browning of stem and root, rapid degradation of cotyledon chlorophyll, no lateral root formation, and necrosis (**Figure 3B**) thereby confirming the inheritance of *bar* gene by the healthy seedlings (PPT resistant) only. The Mendelian principle of 3:1 ratio best fitted (analyzed by Chi square test) for *bar* gene segregation analysis based on ratio of the PPT resistant and PPT sensitive condition of plants (**Table 2**).

**Table 2:** Segregation analysis of transgene (*bar*) in advanced T_2_ generation progeny plants.

Progeny plants	Plant survivability % in PPT (10 mg/l)	PPT resistant	PPT sensitive	PPT resistant: PPT sensitive	Best fit segregation ratio	Chi square value	*P*-value
JBC1.1.1	74.50	149	51	2.92:1	3:1	0.027	0.870
JBC2.1.1	76.00	152	48	3.16:1	3:1	0.107	0.744
JBC3.1.1	72.50	145	55	2.63:1	3:1	0.667	0.414

*Plant survivability % = (number of PPT resistant plants/total plants) × 100. The two-tailed P-value has been represented and Chi square value has been represent with 1 degree of freedom. PPT (phosphinothricin) or glufosinate ammonium or glufosinate, is a herbicide. Here, the three transgenic lines of JBC1, JBC2, and JBC3 have been represented by the individual transgenic T_2_ plants JBC1.1.1, JBC2.1.1, and JBC3.1.1, respectively.*

### Qualitative Analysis of *chi11* Gene

**FIGURE 4:**
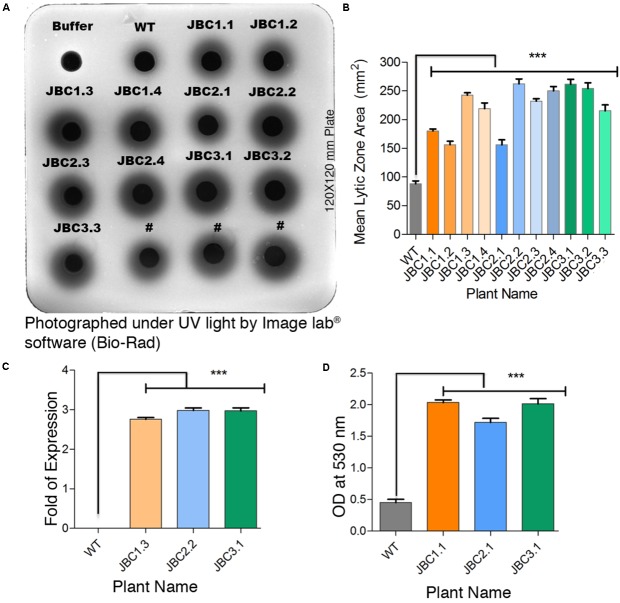
Qualitative analysis specific to *chi11* gene and its functionality. **(A)** Gel diffusion assay- 150 μg crude protein pipetted into individual wells in the 1.5% agarose with 0.4% colloidal chitin gel plate. After incubation, lytic zones visualized by UV transillumination. #Not applicable for investigation. **(B)** A significant difference (^∗∗∗^*P* = < 0.001) in mean zone area (calculated from three replicates) between transgenic and WT plants observed. **(C)** Fold change of developed zone area in transgenic plants over WT represents a significant difference (^∗∗∗^*P* = < 0.001). **(D)** In-solution chitinase assay; 100 μg of total protein incubated with colloidal chitin and the resulting product measured at 530 nm. A significant difference (^∗∗∗^*P* = < 0.001) in OD values between transgenic and non-transgenic WT plant observed. One-way ANOVA was used to compare the means and differences between group means at *P* < 0.05 and Tukey’s multiple comparisons taken under consideration by using Graphpad Prism 6 software. Here specific individual plants are the representatives of the three transgenic lines JBC1, JBC2, and JBC3. A total of 10 biological replicates were analyzed from each transgenic line.

In the gel diffusion assay, the zones hydrolyzed by crude protein extracts were clearly visible under UV light as dark circles surrounding each well (**Figure 4A**). For transgenic plants, zone areas were significantly (*P* < 0.001) 1.77- to 3.08-fold larger than that of non-transgenic (WT) control plants. Mean zone area of T_1_ transgenic plants viz. JBC1.3 (242.66 mm^2^), JBC2.2 (262.33 mm^2^), and JBC3.1 (161.67 mm^2^) showed 2.85-, 3.08-, and 3.23-fold higher chitinase expression than WT plants respectively (**Figures 4B,C**). In the “gray view” of gel using colormap^TM^ tool in image lab^®^ software (Bio-Rad) the zone boundaries were more prominently and clearly visible (**Figure 4A**). For protein extraction buffer (used as a control) no hydrolyzed zone appeared. One-way ANOVA was used to compare the means and differences between group means at *P* < 0.05. A significant difference (i.e., *P* < 0.001) between control and transgenic jute plants was found by the Tukey’s multiple comparison test.

The in-solution chitinase activity assay revealed a significant (*P* < 0.001) increase in enzymatic activity in all T_1_ transgenic plants with respect to non-transgenic or WT. The statistical significance of mean value difference was tested by one-way ANOVA and Tukey’s multiple comparison tests at *P* < 0.05. Reddish-brown coloration of the solution was found for transgenic plants, whereas WT showed a dull yellowish coloration. Among the T_1_ transgenic plants, mean OD values at 530 nm of JBC1.1 (2.04), JBC2.1 (1.72), and JBC3.1 (2.01) were 3.81–4.5 times higher than that of WT plants (0.45) (**Figure 4D**).

### Analysis of Expression of *chi11* and *bar* Genes by Quantitative Real-Time PCR (qRT-PCR)

**FIGURE 5:**
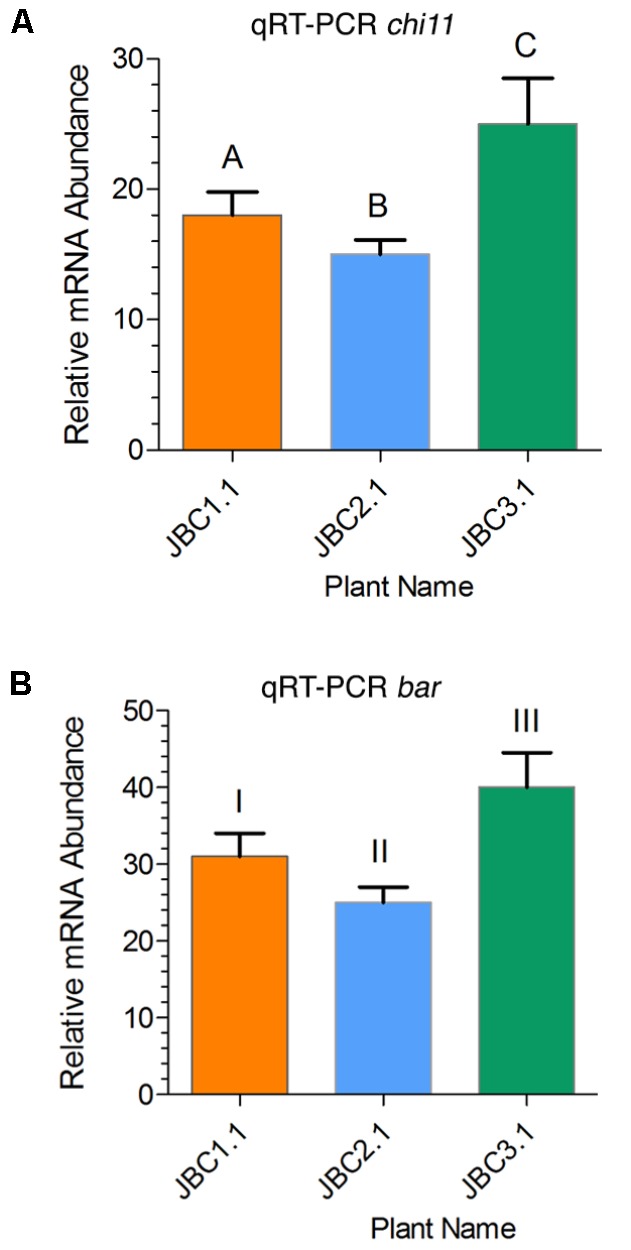
Relative quantities of *chi11* and *bar* mRNA transcripts in transgenic lines as determined by qRT-PCR. **(A)** Relative expression of *chi11* gene in transgenic lines. Graph showing significant difference (in Tukey’s multiple comparisons test) between transgenic lines A-C and B-C = *P* < 0.0001 and A-B = *P* < 0.0014. **(B)** Relative expression of *bar* gene in transgenic lines. Graph showing significant difference between transgenic lines I-III and II-III = *P* < 0.0001 and I-II = *P* < 0.0014 in Tukey’s multiple comparison test analyed by the Graphpad Prism 6 software. Each bar represents the mean ± standard error (SE) of three independent experiments. Here, transgenes expression was calculated on the basis of internal control *26S rRNA* gene expression. Here, the three individual plants JBC1.1, JBC2.1, and JBC3.1 are the representatives of three transgenic lines JBC1, JBC2, and JBC3, respectively. The qRT-PCR analyses were performed with 10 biological replicates from each T_1_ transgenic line with three technical replications for each plant.

In the JBC3.1 transgenic line the expression level of *chi11* and *bar* gene was 25- and 40-fold higher respectively than the level of expression of internal control *26S rRNA* gene (**Figure 5**). A significant difference in gene expression level was also noticed between the progeny plants of these three transgenic lines by the help of one-way ANOVA test. Expression of transgenes, in specific progeny plant (from three transgenic lines), has been represented in **Figure 5**. Significant difference, of *chi11* transcript expression, between transgenic lines JBC1.1 and JBC2.1, JBC2.1 and JBC3.1 = P < 0.0001, JBC1.1 and JBC2.1 = P < 0.0014 was found in the Tukey’s multiple comparison analysis (**Figure 5A**). Significant difference of *bar* transcript expression was found between the transgenic lines JBC1.1 and JBC3.1, JBC2.1 and JBC3.1 = P < 0.0001, JBC1.1 and JBC2.1 = P < 0.0014 (**Figure 5B**). The expression of *chi11* and *bar* genes, in the three transgenic progenies, was found as JBC 3.1 > JBC1.1 > JBC2.1. Non-transgenic (WT) control jute (JRC-321) plants did not show any expression of *chi11* and *bar* genes.

### Herbicide Tolerance Bioassay of Transgenic Plants

**FIGURE 6:**
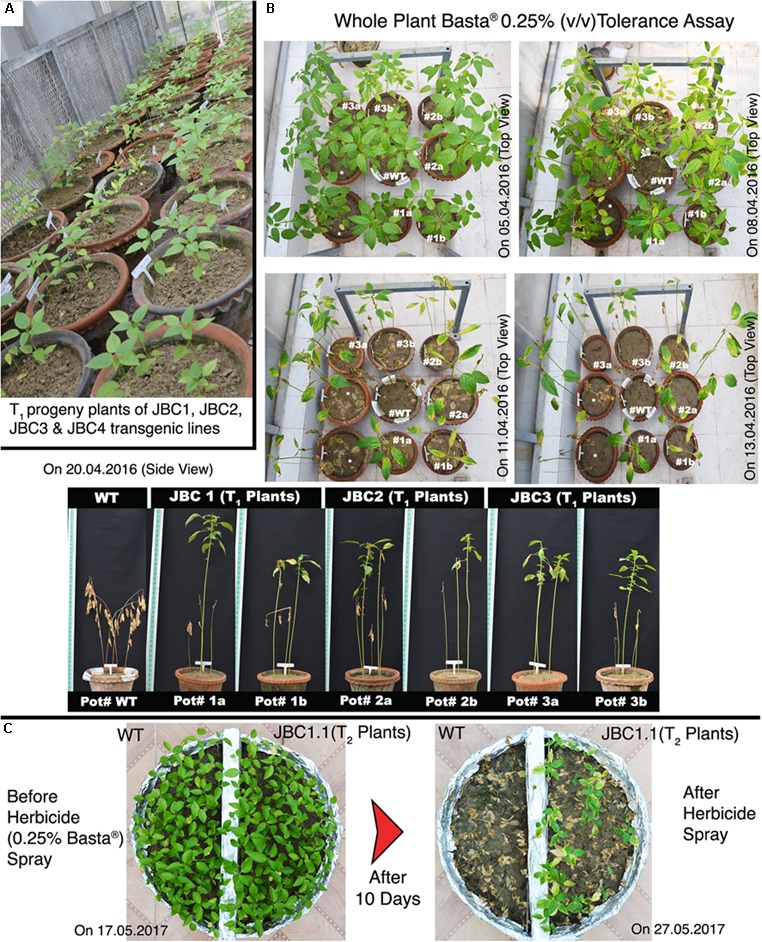
Herbicide (Basta^®^) tolerance ability of transgenic plants. **(A)** T_1_ progeny plants grown in greenhouse condition (progeny plants of JBC4 transgenic line are excluded from investigation). **(B)** In every set, pots of 30 DAS transgenic plants organized by keeping a WT plant in central position. Progenies of three transgenic plant lines showed tolerance to 0.25% (v/v) Basta^®^ but non-transgenic WT plants died early in the 15 day bioassay period. Pots numbered as #tag in photograph with date of picture taken. ^∗^Not applicable for investigation. **(C)** Basta^®^ bioassay in ‘same-pot’ method of T_2_ progeny plants with WT where only the herbicide tolerant transgenic plants survived after the 10 day bioassay period.

The T_1_ progeny plants of the three transgenic lines JBC1, JBC2, and JBC3 were grown and selected for herbicide resistance by subjecting them to the bioassay (**Figure 6A**). The herbicide tolerance bioassay involved spraying of Basta^®^ 0.25% (v/v) on T_1_ progeny plants of 30 DAS old for 10 days and keeping WT as control (**Figure 6B**). Non-transgenic (WT) control plants suffered effects of the herbicide after 12 h and died after 6 days of Basta^®^ spray (**Figure 6B**). Negative transgenic (segregated progeny) plants also showed chlorosis, defoliation, and even death. In transgenic plants a steady recovery from the herbicide stress was noticed from day 7 onwards by the emergence of new leaves and continuous gain in stem length. Progeny plants (at seedling stage) of T_2_ generation were selected again based on their Basta^®^ (0.25% v/v) tolerance (**Figure 6C**).

### Assessment of Phytotoxic Effects of Residual Basta^®^ on Indicator Plants

No negative effect of Basta^®^ herbicide was found on seed germination of indicator plants (cucumber and corn). Cucumber seed germination was 90.00% in untreated pots and 90.00–93.33% in Basta^®^ treated pots. Corn seed germination was 93.33% in untreated pots and 90.00–96.66% in Basta^®^ treated pots (Supplementary Table 2). Seedlings of both indicator plants were healthy in treated and non-treated pots. No phytotoxic effects of residual herbicide was seen on growth of the indicator plants when plant height was measured 2 weeks after sowing (Supplementary Figure 1). Mean height of cucumber plant was 67.55 mm in untreated pots and 70.25, 68.59, and 71.22 mm in 0.25, 0.50, and 1.0% Basta^®^ treated pots respectively. Mean height of corn plant was 326.66 mm in untreated pots and 329.75, 332.51, and 331.67 mm in 0.25, 0.50, and 1.0% Basta^®^ treated pots respectively (Supplementary Table 3). Data derived after mean comparisons of seed germination and plant height were statistically non-significant (*P* ≥ 0.05) for among herbicides, dosages within herbicide and with untreated (water) control sets. According to Pestemer et al. (1980) classification on the effect of residual herbicides on plant, the 0.25–1.0% Basta^®^ treatment was found to be safe for growth of plants.

### Assessment of the Fungus Resistant Ability of Transgenic Plants

**FIGURE 7:**
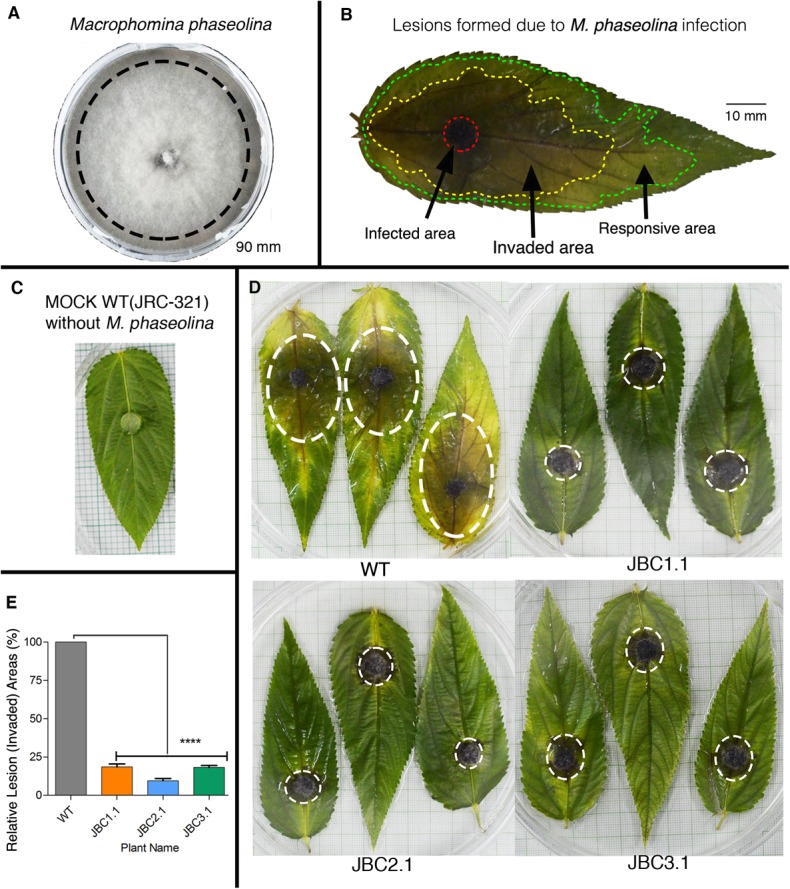
Detached leaf antifungal bioassay of transgenic plants. **(A)** PDA plate of *M. phaseolina* pure culture. Black line indicates the peripheral zone from where 5 mm disks of inoculum was taken for fungal bioassay. **(B)** Schematic diagram of a JRC-321 control jute leaf with three distinct lesion areas (infected, invaded, and responsive area) developed by *M. phaseolina* infection. **(C)** A mock JRC-321 jute leaf (WT without *M. phaseolina* inoculation) bio-assayed with a PDA disk. **(D)** After 72 h of bioassay, reduction in invaded lesion area (white circled) observed in transgenic leaves compared to non-transgenic WT. No such area formed in mock JRC-321 leaf. Experiments replicated three times. **(E)** A significant difference (^∗∗∗∗^*P* < 0.0001) in relative lesion area percentage between transgenic progenies and WT recorded from the one-way ANOVA at *P* < 0.05 and Tukey’s multiple comparisons analysis in Graphpad Prism 6 software. Here, three individual plants JBC1.1, JBC2.1, and JBC3.1 are the representatives of the three transgenic lines JBC1, JBC2, and JBC3, respectively.

After selecting transgenic plants on the basis of herbicide tolerance they were put through a test for fungus resistance against *M. phaseolina* by detached leaf bioassay (DLB) at 40–50 DAS and whole plant bioassay (WPB) at 80–90 DAS of plant age. Pure culture of *M. phaseolina* was used as inoculum for bioassay (**Figure 7A**). In the 72 h of DLB, three distinct areas of lesions were formed on WT leaves due to *M. phaseolina* infection – infected (black/dark brown color), invaded (brown-yellow color) and responsive area (yellow/light green color) (**Figure 7B**). In transgenic leaves lesion areas were comparatively smaller in size than non-transgenic WT jute (**Figure 7D**). In the mock (WT without *M. phaseolina* inoculation) leaf no such areas developed during bioassay (**Figure 7C**). Relative lesion area percentage for transgenic leaves was calculated based on WT leaf where JBC1.1 was 18.58%, JBC2.1 was 9.58%, and JBC3.1 was 18.23% (**Figure 7E**). These data were found to be statistically significant by one-way ANOVA and Tukey’s multiple comparison tests at *P* < 0.05 (**Figure 7E**). It was also observed that lesion formation in transgenic leaves slowed down after 48 h of inoculation but in the case of WT lesion area formation continued even after 72 h.

**FIGURE 8:**
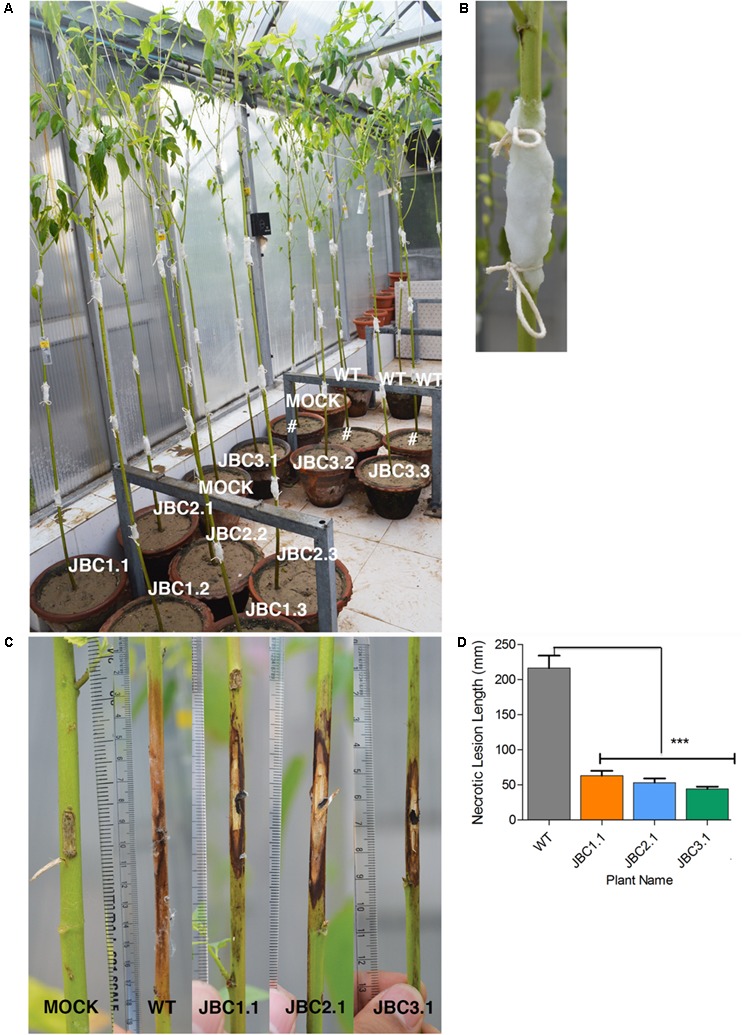
Whole plant antifungal bioassay of transgenic plant. **(A)** Representative picture of whole plant *M. phaseolina* bioassay setup in greenhouse condition. Progenies of 4 T_1_ transgenic lines, non-transgenic WT and non-transgenic mock (WT without fungal inoculation) plants of 80–90 DAS arranged with replicates. #Not applicable for investigation. **(B)** Stem pricked with sterile scalpel in 3–4 positions at equal intervals from soil surface and 5 mm mycelia disk of *M. phaseolina* inoculum placed on it and covered with moist cotton. **(C)** Representative picture of necrotic lesion formation due to infection. Lesion length (mm) measured which was larger in WT than transgenic progenies and no necrotic lesion formed in mock plants. **(D)** Significant difference (^∗∗∗^*P* < 0.001) found in mean lesion length between transgenic progenies and WT by one-way ANOVA at *P* < 0.05 and Tukey’s multiple comparisons analysis. Here, specific individual plants are the representative of the three transgenic lines JBC1, JBC2, and JBC3.

In WPB, necrotic lesion formed on plant stem due to *M. phaseolina* infection (**Figure 8**). The mock plants completely recovered from the initial injury inflicted for inoculation and no necrotic lesion developed during the 20 days bioassay period (**Figure 8C**). A significant difference (*P* < 0.001) in mean lesion length between transgenic and non-transgenic (WT) plants was found after bioassay in one-way ANOVA and Tukey’s multiple comparison tests (at *P* < 0.05). In WT it was 216.66 mm and in transgenic progenies JBC1.1 it was 63.3 mm, in JBC2.1 it was 53.3 mm and in JBC3.1 it was 44.3 mm (**Figure 8D**). The longest lesion found for WT plants measured 400 mm in length and that for transgenic plants it was 230 mm as observed during whole plant antifungal bioassay.

### Agronomical Comparative Analysis of FR-HT Transgenic Jute and WT Jute Plants

The agronomic variables, of plant height, stem length, and basal stem diameter of transgenic jute and WT jute plants of ages 110–120 DAS, were at par (**Table 3**). No significant difference was observed in agronomic traits between T_2_ FRHT jute and non-transgenic jute plants (*P* ≥ 0.05).

**Table 3:** Comparative analysis of agronomic characters of non-transgenic (WT) and transgenic T_2_ plant progenies in greenhouse condition.

Agronomic trait	WT	JBC1	JBC2	JBC3
Plant height (m)	3.22 ± 0.10	3.10 ± 0.18	3.30 ± 0.17	3.25 ± 0.18
Stem length (m)	3.00 ± 0.12	2.91 ± 0.16	3.11 ± 0.13	2.97 ± 0.12
Basal stem diameter (mm)	21.00 ± 1.1	20.50 ± 1.8	19.80 ± 1.54	21.20 ± 0.98
		n.s.	n.s.	n.s.

*The values represent the mean data of 10 progeny plants (*n* = 10) from each line ± standard error. Mean comparisons of different agronomic traits between WT and transgenic jute lines and between the three transgenic lines were non-significant (n.s.) with P ≥ 0.05 (in ANOVA test).*

### Assessment of Fiber Quality for *M. phaseolina* Resistant Transgenic Plants

**FIGURE 9:**
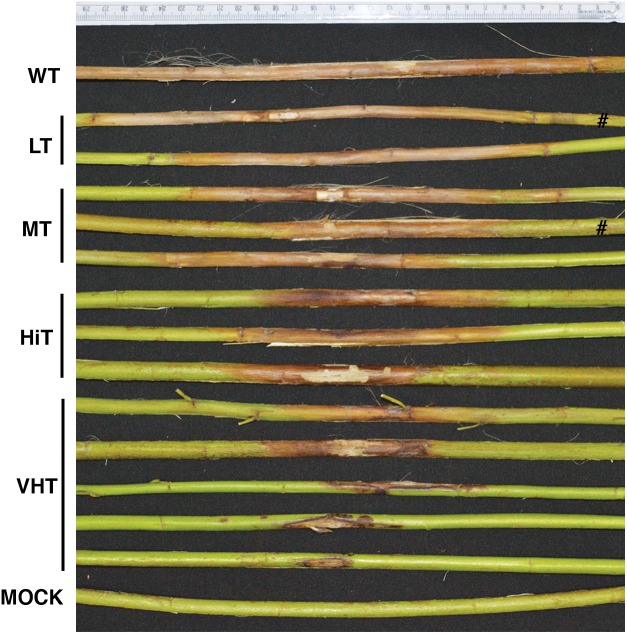
Different levels of antifungal activity observed after bioassay of transgenic progeny plants. Plants categorized on the basis of developed lesion length as very high tolerance (VHT), high tolerance (HiT), moderate tolerance (MT), and low tolerance (LT). #Not applicable for investigation.

**FIGURE 10:**
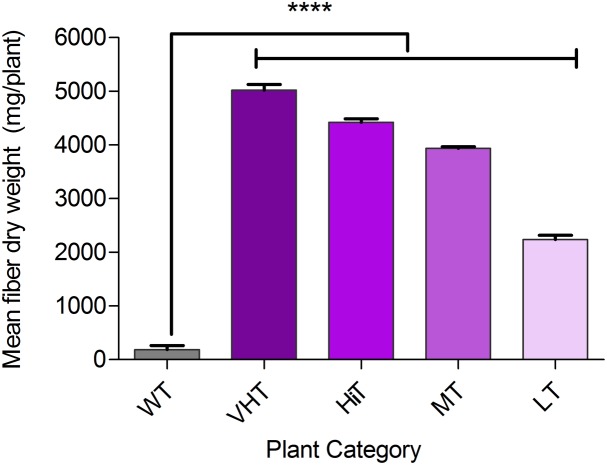
Bast fiber yield (dry fiber weight per plant) under *Macrophomina phaseolina* infected condition in greenhouse. Plant categorized on the basis of developed lesion length as non-transgenic control (WT), VHT, HiT, MT, and LT transgenic jute plants. Fibers were extracted from 10 progeny plants (120–130 DAS) of each category, sun dried and recorded for dry weight (mg/plant). The values represent the mean of fiber dry weight ± standard error (*n* = 10). Significant difference (^∗∗∗∗^*P* < 0.0001) found in fiber production between transgenic progenies and WT by one-way ANOVA at *P* < 0.05 and Tukey’s multiple comparisons analysis.

Effect of *M. phaseolina* infection is directly related to fibre yield and quality losses as analyzed by selecting 10 transgenic plants from each category viz. VHT (lesion length 10–100 mm), HiT (101–130 mm), MT (131–170 mm), and LT (171–230 mm) (**Figure 9**). It was observed that LT category of transgenic plants retted comparatively faster (within 6–8 days). Retting time for VHT, HiT, and MT category transgenic plants was 10–12 days. Mock JRC-321 (without fungal inoculation) non-transgenic plants required 12 days time for proper retting. A non-significant difference (*P* ≥ 0.05) in fiber length and strength was found between mock JRC-321 control and VHT and HiT category plants in the ANOVA statistical analysis (**Table 4**). The fiber length (1.41 m) and strength (10.1 g/tex) was found to be reduced by about 50% in LT category plants compared to control. The quality of fiber was similar (about 1.5 tex) in all tested plants and non-significantly varied (*P* ≥ 0.05) in test categories (as analyzed by ANOVA). Fiber yield was calculated from the dry weight of fibers (mg/plant) and significant difference (*P* < 0.0001) was found between VHT, HiT, MT, and LT categories after analysis by ANOVA (one-way) and Tukey’s multiple comparison statistical tests (**Figure 10**). Good amount of fiber extraction was not possible from the *M. phaseolina* infected WT plants that led to about 100% fiber yield loss.

**Table 4:** Comparative analysis of fiber quality of mock WT (non-transgenic) control and *Macrophomina phaseolina* resistant transgenic T_1_ progeny plants.

Fiber qualities	Mock WT	*M. phaseolina* resistant transgenic jute (category wise)			
		Very high tolerance (VHT) plants	High tolerance (HiT) plants	Moderate tolerance (MT) plants	Low tolerance (LT) plants
Fiber length (m)	3.00 ± 0.30	3.00 ± 0.31 n.s.	2.8 ± 0.20 n.s.	2.55 ± 0.16	1.11 ± 0.30
Fiber strength (g/tex)	22.1 ± 2.11	21.6 ± 1.20 n.s.	21.1 ± 2.81 n.s.	16.2 ± 1.66	10.1 ± 2.09
Fiber fineness (tex)	1.50 ± 0.006	1.49 ± 0.002 n.s.	1.50 ± 0.001 n.s.	1.48 ± 0.007 n.s.	1.47 ± 0.002

*Mock WT jute is non-transgenic control without *M. phaseolina* inoculation. The values represent the mean of 10 progeny plants from each category ± standard error. Mean comparisons with WT control are non-significant (n.s.) with P ≥ 0.05 (in ANOVA test).*

## Discussion

The demand for jute fibers and its applications are ever increasing mainly due to its versatile usage and environment friendly nature. The supply of jute fibers, to meet this worldwide demand, is done by a few Asian countries that cultivate jute plants. Farmers of these developing countries face endless biotic, abiotic, and financial challenges to maintain a healthy jute production. Along with many other adverse factors, the cost of cultivation increases when jute fields need management to alleviate infestation of weeds, fungi and insects. Protection from devastating Lepidoptera, that devour jute plants, was achieved when Majumder et al. (2018) first developed transgenic Bt-jute and successfully tested it against major insect pests of jute. It assured healthy jute production and fiber quality. In this study, we have developed a fungus resistant (FR)- herbicide tolerant (HT) transgenic jute that holds the promise of maintaining the yield and quality of fiber even in a fungus and weed infested field. We preferred the JRC-321 variety for developing FRHT jute due to its ability to produce finest quality fibers (1.5 tex) with high yield (20–25 quintal/hectare). It is suitable for jute-cotton blend yarn, fabric, and textile production^6^.

The non-selective herbicides glyphosate (Roundup^®^) and glufosinate (Basta^®^) are predominantly used for weed management in agricultural fields. Extensive field application, of any single herbicide repeatedly, can create a selection pressure on weeds and induce them to attain resistance against it. Glyphosate resistance was first reported in 1996 in Australia and now 38 species of weeds have evolved resistance to it, distributed across 37 countries and in 34 different crops (Heap and Duke, 2018). In present days, glyphosate-resistant weeds are the greatest threat to sustained weed control in major agronomic crop fields. This problem can be solved by using herbicides with a different mode of action or creating transgenic crops with multiple herbicide resistant genes (Duke, 2005; Green, 2014). Glyphosate-resistant weeds in the fields can be controlled by glufosinate herbicides. A recent study validated this strategy of weed management and found it to be highly effective and economical for control of glyphosate-resistant weeds in a glufosinate-resistant transgenic soybean field in Gage County, NE, United States (Barnes et al., 2017). The occurrence of glyphosate-resistant weeds in cotton fields has been reported worldwide^7^. India, Bangladesh, and China cultivate both cotton (including glyphosate-resistant cotton) and jute for fiber production. Therefore in case of infestation by glyphosate-resistant weeds in jute fields, glufosinate herbicide could be effective for weed management.

Glufosinate herbicides, including Basta^®^, have been well tested on a variety of weeds and crops. It has been reported by Abdeen and Miki (2009) that, after 6 to 48 h of spraying Basta^®^ herbicide, *Arabidopsis* control plants endured inhibition of glutamine synthetase (GS) downstream metabolic pathways. This led to inhibition of photosynthesis and leaf senescence-related processes ultimately leading to plant death. Transgenic *Arabidopsis* plants with *bar* gene survived these adversities. In our study, Basta^®^ adversely affected non-transgenic WT jute plants after 24 h of spraying and caused death within 6 days (**Figure 6**). After treatment with PPT, ammonia accumulated in the plant tissues which led to death, as detected in the CPR assay (**Figure 2A**) for WT jute plants. The deposited ammonia diffused from the non-transgenic jute tissues and helped to develop the blue color in AA assay (**Figure 2D**). The FRHT transgenic jute plants produced PAT enzyme, as detected in ELISA (**Figure 2E**). PAT catalyzes the acetylation of L-PPT to produce *N*-acetyl-L-PPT thereby rendering it unable to inactivate GS of the FRHT jute. The catalytic activity of PAT is specific for L-PPT in the presence of co-substrate acetyl-CoA (Wehrmann et al., 1996). The *bar* gene and its PPT detoxifying ability makes it a potent genetic marker, commonly used in many plant genetic transformation events in addition to HT crop development (Vasconcelos et al., 2003; Miki and McHugh, 2004). Basta^®^ selection process was effective in identifying high expressive positive jute plants from a large population of plants in a short time.

Glufosinate herbicides (Basta^®^) have some advantages. They can be mixed with other herbicides during field application. Unlike glyphosate herbicides (Roundup^®^), they can be used throughout the growing season at all crop growth stages and can be used in seasonal applications^8^. Basta^®^ is very low in toxicity to humans and animals compared to other herbicides. It is absorbed by the organic particles in the soil and has a short half life thereby decomposing rapidly. It poses little danger by leaching and contamination of ground water or toxicity to wildlife (Duke, 2005). We found that 0.25 to 1.0% Basta^®^ has no residual phytotoxic effect on the indicator plants -corn and cucumber when compared with those from untreated (control) pots. Germination rate and seedling development of the indicator plants were more than 90% normal (Supplementary Table 2). Wibawa et al. (2009), reported glufosinate herbicides to have no residual phytotoxic effect on indicator plants (corn and cucumber), even in high doses (200 to 800 g a.i./hectare).

In T_1_ and T_2_ generations of FRHT jute, we extensively used the function of *bar* gene (as a herbicide marker) against Basta^®^ (13.5% PPT) in order to select positive plants. Optimization of three different glufosinate doses was achieved to develop and select FRHT jute (JRC-321) plants. For shoot tip (explant) selection the concentration of PPT was 4 mg/l (w/v), for seedlings 10 mg/l (w/v) PPT and for whole plant 0.25% (v/v) Basta^®^ herbicide. Shoot tips were selected by three consecutive selections at fortnightly intervals by gradually increasing the PPT concentration in media from 1 to 4 mg/l. This approach of gradually increasing the concentration of PPT ensures the survival of the shoot tips. Alternatively, subjecting shoot tips abruptly to a PPT concentration of 4 mg/l led to lethal effect on shoot regeneration. Latha et al. (2006), used four stages of selection, i.e., 3, 5, 8, and 12 mg/l of PPT and finally used 0.20% Basta^®^ for selecting pearl millet containing the *bar* gene. Using a needlessly high dose is fatal for untransformed tissues and inhibits growth of transformed cells thereby delaying the process of regeneration (Wilmink and Dons, 1993). We applied gradient doses of Basta^®^ on some randomly selected grassy and broadleaved weeds (from jute fields) and found 0.20% (v/v) to be the effective dose for majority of them (Supplementary Figure 2). Based on this observation FRHT transgenic jute plants were selected by spraying a higher dose of 0.25% (v/v) Basta^®^. In this investigation, we selected such sub-lethal doses where only transgenic plants could survive without compromising on growth (**Figures 3**, **6**). Similar strategy, for selecting other transgenic crops having *bar* with/without *chitinase* gene(s), has been reported by Naing et al. (2016) where 1.0 mg/l PPT and 0.20% Basta^®^ was used for chrysanthemum with *bar* gene. Chen et al. (1998) reported the use of 5.0 mg/l PPT and 0.20% Liberty for wheat with *OsChi11* and *bar* genes. Datta et al. (1992) used 20 mg/l PPT and 2.0% Basta^®^ for selection of transgenic rice with *bar* gene. Previous reports on jute transformation (biolistic) of JRC-321 mentioned use of *bar* gene as selectable marker where transgenic plants were selected in 2.0 to 2.5 mg/l PPT (Bhattacharyya et al., 2015). Up to April 2018, a total of 233 transgenic events of glufosinate – tolerant crops have been registered for canola, chicory, cotton, rice, maize, soybean, and sugar beet according to ISAAA’s ‘GM approval database’^9^. Many of these crops have been commercially cultivated for more than 20 years in the United States and worldwide with high economic benefits (Duke, 2015).

Transgenic jute plants were tested against its most devastating necrotrophic fungal pathogen – *M. phaseolina*. The stem rot causing property of the pure culture of *M. phaseolina* was successfully tested on JRC 412, a susceptible variety of *C. capsularis* and then used in this study (Biswas et al., 2014). We found that *C. capsularis* variety JRC-321 has no resistance against *M. phaseolina* infection. Large lesions were observed in non-transgenic (control) jute plants within 48 h of infection and within 72 h leaves were completely damaged (**Figure 7**). Plant stems were inoculated at 3–4 positions, at equal length intervals from soil, to increase the fungal load on each plant under WPB (**Figure 8A**). Necrotic lesions covered an entire non-transgenic (control) jute plant within 15–20 days (**Figure 9**). We observed that stems of young JRC-321 plants (up to 65–75 DAS) were barely affected by *M. phaseolina* infection but leaves of all ages showed lesions. This may be due to the fact that *M. phaseolina* (necrotrophic pathogen) requires dead tissue for establishment of infection which was available in the mature jute stems. This is similar to the rice blast fungus *Magnaporthe oryzae* that requires young (living) plant tissues during the early stages of infection (Okagaki and Dean, 2016). Based on this observation we checked the antifungal activity of transgenic plants of 80–90 DAS age. At this age fiber growth is on its peak and any stem affecting disease can cause serious reduction of jute fiber yield and quality. The *M. phaseolina* infected plants exhibited better fiber retting ability than others. This may be due to the presence of hydrolytic enzymes (cellulase and hemicellulase) produced by the fungus during host infection, that weakens the bonding between fiber tissue and stem of jute plant (Islam et al., 2012).

Plants naturally produce chitinase in response to various stress stimuli (Collinge et al., 1993). Chitinase is an integral part of the plant defense system, primarily against fungal pathogen (Boller, 1987). *In vitro* studies have demonstrated a growth inhibitory effect of chitinase by hydrolysis of the apex of growing hyphae, that are made of chitin and β-1,3-glucan fiber (Collinge et al., 1993). Hyphae degrading ability of rice chitinase (*chi11*) enzyme has been reported in transgenic rice against *Rhizoctonia solani* (Karmakar et al., 2015). Inhibition of *M. phaseolina* growth by rice-chitinase enzyme can be visualized in **Figures 7**, **8** for transgenic jute plants. Gel diffusion assay revealed that non-transgenic WT plants also produce chitinase in response to *M. phaseolina* infection (**Figure 4A**). It has been confirmed by antifungal bioassay that the amount of chitinase expression by WT jute plants is not sufficient to develop resistance against *M. phaseolina* (WT in **Figures 7**, **8**). Transgenic expression of rice *chi11* gene in response to the pathogen in jute increases chitinase amount up to 3.23-fold higher than natural level and holds the credit of antifungal activity (**Figure 4C**). This strategy can be utilized to defend other disease causing pathogens of jute like *Glomerella cingulata, Botryodiplodia theobromae, R. solani, Fusarium solani, Pseudomonas solanaceaum, Sclerotium rolfsii, Diplodia corchori, Corynespora cassiicola* or against group of *Colletotrichum* fungi (*C. chorchorum, C. gloeosporioides, C. fructicola, C. siamense*, and *C. corchorum-capsularis*). These pathogens cause huge fiber yield losses in India, Bangladesh, and China (Wadud and Ahmed, 1962; Bhattacharya, 2013; Niu et al., 2016).

The mechanism of host–pathogen interaction in relation to jute and *M. phaseolina* is not well-known and very limited information has been reported. Islam et al. (2012), sequenced 92.83% genome of this fungus and found abundance of hydrolytic enzymes for degrading cell wall component to penetrate into the host tissue. Sarkar et al. (2014), reported that *M. phaseolina* invade plant defenses by expressing high amounts of nitric oxide and reactive nitrogen species along with lowering of reactive oxygen species in plant cells. This leads to leaf yellowing and wilting followed by death.

Development of *M. phaseolina* resistant transgenic plants, developed by parasite-derived resistance (PDR) or RNA silencing technique is yet to be accomplished due to scarcity of information about host–pathogen interaction. Similarly, approaches like host-induced gene silencing (HIGS) for jute plant is highly challenging due to dearth of scientific knowledge in this field. Change in genetic variability by conventional breeding is immensely difficult due to a strong sexual incompatibility barrier between jute species. Therefore development of transgenic jute plants, expressing chitinase, is an effective strategy for improving the plant defense mechanism against chitin containing fungal pathogens including *M. phaseolina*. An initial report on draft jute genome sequencing data indicated large number of disease resistance-like genes present in jute, which could be utilized in future for transgenic development (Sarkar et al., 2017).

Jute is known for its fibers of versatile use. With the steady increase of demand for natural fibers in the world, jute is now considered as a “future fiber” crop^10^. We analyzed fiber yield and quality of *M. phaseolina* infected jute plants in greenhouse conditions. Long intact fibers could not be recovered from WT control plants due to complete necrosis of stem tissue. It was found that stem rot causes about 100% fiber loss in non-transgenic jute plants along with reduction in fiber quality (**Figure 10**). As the study was conducted in greenhouse, actual ‘jute cultivation cost’ analysis for field condition was outside the scope of this study.

These FRHT transgenic jute lines have the potential to reduce total cultivation cost by minimizing expensive manual weed management (large manpower needed), reducing the use of chemical fungicides and reducing the number of selective herbicide sprays in a season. In other words, farmer’s income will increase by minimizing investment and they will reap benefits of higher fiber quality and yield even in *M. phaseolina* infected fields. This first contribution toward fungus (*M. phaseolina*) resistant and herbicide tolerant transgenic jute plant development holds tremendous scientific and agricultural impact on society.

## Author Contributions

SD and KD designed the experiments. SM planned this research, conducted the experiments, and prepared the manuscript. CS assisted in carrying out the experiments. SM, CS, KD, and SD performed the data analyses. SS performed fiber quality assesments. SD, KD, and SS edited the manuscript. All authors discussed the results and commented on the manuscript.

## Conflict of Interest Statement

The authors declare that the research was conducted in the absence of any commercial or financial relationships that could be construed as a potential conflict of interest.

## Footnotes

1 http://faostat3.fao.org

2 www.isaaa.org/gmapprovaldatabase/geneslist

3 https://blast.ncbi.nlm.nih.gov/Blast.cgi

4 graphpad.com

5 https://www.graphpad.com/quickcalcs/chisquared1.cfm

6 jafexpert.crijaf.icar.gov.in

7 http://www.weedscience.org

8 www.crop.bayer.com.au

9 www.isaaa.org/gmapprovaldatabase/default.asp

10 http://www.fao.org/economic/futurefibres/home/en/
