# Supplementary material for: The Development of Macrophomina phaseolina (Fungus) Resistant and Glufosinate (Herbicide) Tolerant Transgenic Jute
Source: Front Plant Sci. 2018 Jul 10;9:920. doi: 10.3389/fpls.2018.00920 (PMC6048421; doi:10.3389/fpls.2018.00920)
Supplement: Supplementary file 1 [file Table_1.pdf]

frontiers in Plant Science
ORIGINAL RESEARCH
published: xx June 2018
doi: 10.3389/fpls.2018.00920

Majumder S, Datta K, Sarkar C, Saha SC and Datta SK (2018) The Development of *Macrophomina phaseolina* (Fungus) Resistant and Glufosinate (Herbicide) Tolerant Transgenic Jute. *Front. Plant Sci.* 9:920. doi: 10.3389/fpls.2018.00920

Supplementary Table 1

Summary of the transformation experiments using shoot tip explants of *C. capsularis* cv. JRC 321

Set	Number of explants used	Number of plants grown in green house	Number of *bar* and *chi11* PCR positive plants	Transformation efficiency (%)	Mean transformation efficiency (%)
1	158	3	3	1.90	
2	143	3	3	2.10	3.00
3	200	4	4	2.00	

The 3 transgenic plants that we have reported were out of 10 T_0 transgenic lines. These 3 transgenic plants (named as JBC1, JBC2 and JBC3) have been mentioned separately because they showed the highest expression of transgene in qRT-PCR out of the aforementioned 10 lines. They showed better recovery after Basta® whole plant bioassay, tested positive for antifungal activity under bioassay and produced ample seeds as compared to the other 7 transgenic T_0 transformants.
