# Supplementary material for: The Development of Macrophomina phaseolina (Fungus) Resistant and Glufosinate (Herbicide) Tolerant Transgenic Jute
Source: Front Plant Sci. 2018 Jul 10;9:920. doi: 10.3389/fpls.2018.00920 (PMC6048421; doi:10.3389/fpls.2018.00920)
Supplement: Supplementary file 2 [file Table_2.pdf]

frontiers in Plant Science

ORIGINAL RESEARCH
published: xx June 2018
doi: 10.3389/fpls.2018.00920

Majumder S, Datta K, Sarkar C, Saha SC and Datta SK (2018) The Development of *Macrophomina phaseolina* (Fungus) Resistant and Glufosinate (Herbicide) Tolerant Transgenic Jute. *Front. Plant Sci*. 9:920. doi: 10.3389/fpls.2018.00920

Supplementary Table 2

Effect of Basta® herbicide residues (in soil) on Germination percentage of indicator plants (cucumber and corn)

Basta® herbicide % (v/v)	Mean germination percent 6 days after herbicide spray	
	Cucumber (%)	Corn (%)
Untreated (Water)	90.00	93.33
0.25% Basta®	93.33	96.66
0.50% Basta®	86.66	96.66
1.00% Basta®	90.00	90.00
	n. s.	n. s.

Mean percent germination = (Total number of germinated seedlings / Total number of seeds) X 100. Cumulative data set of 3 replications have been represented. Mean comparisons are non-significant (n.s.) at $P<0.05$ among herbicide, doses within herbicide and with untreated (water) control.
