# Supplementary material for: The Development of Macrophomina phaseolina (Fungus) Resistant and Glufosinate (Herbicide) Tolerant Transgenic Jute
Source: Front Plant Sci. 2018 Jul 10;9:920. doi: 10.3389/fpls.2018.00920 (PMC6048421; doi:10.3389/fpls.2018.00920)
Supplement: Supplementary file 3 [file Table_3.pdf]

frontiers in Plant Science

ORIGINAL RESEARCH
published: xx June 2018
doi: 10.3389/fpls.2018.00920

Majumder S, Datta K, Sarkar C, Saha SC and Datta SK (2018) The Development of *Macrophomina phaseolina* (Fungus) Resistant and Glufosinate (Herbicide) Tolerant Transgenic Jute. *Front. Plant Sci*. 9:920. doi: 10.3389/fpls.2018.00920

Supplementary Table 3

Height of indicator plants (cucumber and corn) affected by Basta® herbicide residues in soil

Basta® herbicide % (v/v)	Mean plant height (mm)	
	Cucumber	Corn
Untreated (Water)	70.55 ± 5.55 (n=27)	329.66 ± 25.94 (n=28)
0.25% Basta®	66.25 ± 7.32 (n=28)	326.75 ± 20.10 (n=29)
0.50% Basta®	69.59 ± 5.55 (n=26)	332.51 ± 22.25 (n=29)
1.00% Basta®	71.22 ± 6.12 (n=27)	331.67 ± 26.11 (n=27)
	n. s.	n. s.

Here, n = total number of germinated seedling from sets of 3 replications. Mean comparisons are non-significant (n.s.) at $P<0.05$ among herbicide, doses within herbicide and with untreated (water) control.
