# Supplementary material for: The Development of Macrophomina phaseolina (Fungus) Resistant and Glufosinate (Herbicide) Tolerant Transgenic Jute
Source: Front Plant Sci. 2018 Jul 10;9:920. doi: 10.3389/fpls.2018.00920 (PMC6048421; doi:10.3389/fpls.2018.00920)

frontiers in Plant Science

ORIGINAL RESEARCH
published: xx June 2018
doi: 10.3389/fpls.2018.00920

Majumder S, Datta K, Sarkar C, Saha SC and Datta SK (2018) The Development of *Macrophomina phaseolina* (Fungus) Resistant and Glufosinate (Herbicide) Tolerant Transgenic Jute. *Front. Plant Sci.* 9:920. doi: 10.3389/fpls.2018.00920

Supplementary Figure 1

Effects of residual Basta® on growth (height) of indicator plants cucumber and corn

Indicator plants (cucumber and corn) after two weeks of seed germination in 0.25 %, 0.50 % and 1.0 % Basta sparayed pots with untreated pots as control. Plants were mainained in greenhouse condition and watered regularly.

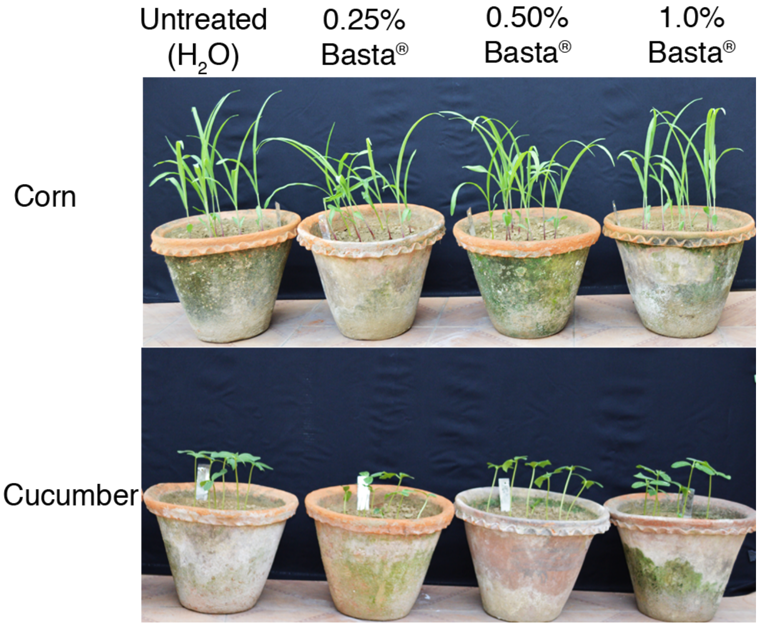

Supplement: Supplementary file 4 [file Image_1.pdf]
