# Supplementary material for: The Development of Macrophomina phaseolina (Fungus) Resistant and Glufosinate (Herbicide) Tolerant Transgenic Jute
Source: Front Plant Sci. 2018 Jul 10;9:920. doi: 10.3389/fpls.2018.00920 (PMC6048421; doi:10.3389/fpls.2018.00920)

frontiers in Plant Science

ORIGINAL RESEARCH
published: xx June 2018
doi: 10.3389/fpls.2018.00920

Majumder S, Datta K, Sarkar C, Saha SC and Datta SK (2018) The Development of *Macrophomina phaseolina* (Fungus) Resistant and Glufosinate (Herbicide) Tolerant Transgenic Jute. *Front. Plant Sci.* 9:920. doi: 10.3389/fpls.2018.00920

## Supplementary Figure 2

### Effects of Basta® herbicide on weeds from jute fields

Grassy and broadleaved weeds were randomly selected from jute fields. Basta® herbicide (0.20 % v/v) was sprayed once on them. Weeds were monitored for 14 days in greenhouse condition. Photograph was taken after 14 days of bioassay.

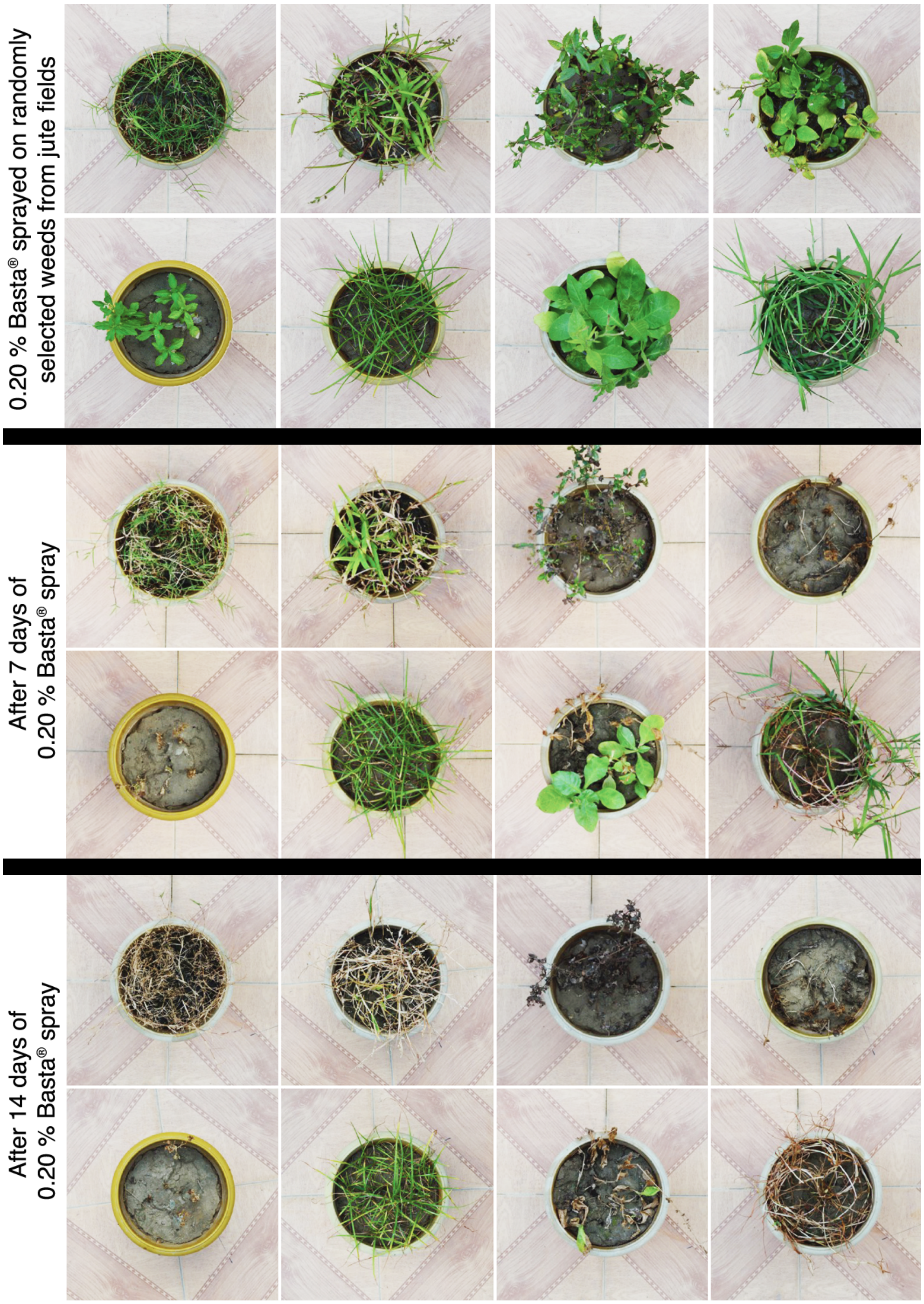

Supplement: Supplementary file 5 [file Image_2.pdf]
